# Genome sequencing and *de novo* assembly of the giant unicellular alga *Acetabularia acetabulum* using droplet MDA

**DOI:** 10.1038/s41598-021-92092-4

**Published:** 2021-06-17

**Authors:** Ina J. Andresen, Russell J. S. Orr, Anders K. Krabberød, Kamran Shalchian-Tabrizi, Jon Bråte

**Affiliations:** 1 Centre for Integrative Microbial Evolution (CIME) and Centre for Epigenetics, Development and Evolution (CEDE), Department of Biosciences, University of Oslo, Kristine Bonnevies Hus, Blindernveien 31, 0316 Oslo, Norway; 2 Natural History Museum, University of Oslo, Oslo, Norway; 3 Section for Genetics and Evolutionary Biology (EVOGENE), Department of Biosciences, University of Oslo, Oslo, Norway; 4 Department of Virology, Norwegian Institute of Public Health, 0213 Oslo, Norway

**Keywords:** Bioinformatics, Genomics, Nuclear organization, Cell biology, Computational biology and bioinformatics

## Abstract

The macroscopic single-celled green alga *Acetabularia acetabulum* has been a model system in cell biology for more than a century. However, no genomic information is available from this species. Since the alga has a long life cycle, is difficult to grow in dense cultures, and has an estimated diploid genome size of almost 2 Gb, obtaining sufficient genomic material for genome sequencing is challenging. Here, we have attempted to overcome these challenges by amplifying genomic DNA using multiple displacement amplification (MDA) combined with microfluidics technology to distribute the amplification reactions across thousands of microscopic droplets. By amplifying and sequencing DNA from five single cells we were able to recover an estimated ~ 7–11% of the total genome, providing the first draft of the *A. acetabulum* genome. We highlight challenges associated with genome recovery and assembly of MDA data due to biases arising during genome amplification, and hope that our study can serve as a reference for future attempts on sequencing the genome from non-model eukaryotes.

## Introduction

During the last decade, developments in DNA sequencing technology have led to a surge in the number of eukaryotic genomes being published. The bulk of these genomes belong to animals, plants, and fungi, while single-celled eukaryotes (protists) remain largely absent^1^. This is unfortunate as protists have an enormous diversity of cellular morphologies, physiology, and genetics, possibly even more so than their multicellular relatives^2^. Although there has been a recent increase in the number of available protist genomes e.g.^3–5^, some groups are still completely devoid of any genomic information^6^.

One protist group, of which we have no genomic information is the green algal order Dasycladales whose species have a very characteristic cellular morphology. Despite being unicellular, and having only a single nucleus, some species can grow to more than 10 cm in length^7^. *Acetabularia acetabulum* is the most studied species of Dasycladales. This umbrella-looking organism is elongated in an apical-basal direction with the root-like rhizoid in the basal end and a disc-shaped cap in the apical end, separated by a long stalk^7,8^.

The size and highly elaborate cellular morphology, together with a large and distinct nucleus, made *Acetabularia* an attractive model system for studies of cell biology and genetics. Already in the 1930s, Joachim Hämmerling used *Acetabularia* to prove that cellular morphogenesis was influenced by so-called “morphogenetic substances” (later confirmed to be RNA) which were produced by the nucleus and distributed to the rest of the cell^9^. By transplanting and exchanging the apical and basal parts between *A. acetabulum* and *A. crenulata*, he observed that the cap developed into the morphology of the basal donor, demonstrating that the nucleus-containing rhizoid was in control of the morphogenetic fate of the cell^10,11^.

Despite its popularity and importance in early cell biology and genetics, the interest in *A. acetabulum* and its sister species dropped towards the end of the 1990s. As of yet, no attempt has been reported to sequence and assemble the genome of *A. acetabulum*, or any other dasycladalean species. The lack of Dasycladalean genomes, and protist genomes in general, can to a large extent be explained by the challenge of obtaining sufficient levels of genomic DNA required for sequencing. The *A. acetabulum* cell has a life cycle of 3 months when cultivated in a highly nutritious media^12^ and cultures cannot be grown densely (maximum 25 algae in 50 ml)^11^. Typical library preparation protocols for whole genome sequencing depend on several hundred nanograms of input DNA, which equates to thousands of *A. acetabulum* individuals for a single sequencing sample. Considering the potentially enormous size of the *A. acetabulum* genome, with the diploid nuclear genome estimated to be 1.85 pg (ca. 1.8 Gb) based on flow-cytometry measurements^13^, this further increases the demand for DNA input.

In order to solve the challenges of limited DNA material, several methods for amplification of genomic DNA have been developed. The earliest whole genome amplification (WGA) methods were based on short-length PCR amplifications with random or degenerate primers^14,15^. These methods often recovered only small fractions of the genomes and were hugely influenced by biases introduced by PCR amplification^16,17^. The most promising development in WGA has been the use of multiple displacement amplification (MDA). This method utilizes the phi29 polymerase which copies DNA with high fidelity and incorporates more than 70,000 nucleotides without falling off the template, resulting in large stretches of amplified DNA^16,18^. However, there are several challenges associated with the phi29-based MDA method. First, as the MDA method relies on random priming, the priming and amplification do not distinguish between target and possible contaminant DNA in the sample. *De novo* assemblies can therefore be challenging if databases lack target genomes or contaminant sequences^19^. Second, and again like PCR-based amplification methods, MDA is also prone to amplification bias. Observations made on bacterial genomes amplified by MDA have shown that certain genomic regions seem to be more readily amplified than others, creating highly uneven coverage across the genome^19–22^. MDA-generated data therefore rarely results in full genome recovery. López-Escardó et al.^23^ used MDA to amplify the genome of three cells of the protist *Monosiga brevicollis* and showed highly uneven coverage and a genome recovery of 6–36% from each cell when mapping to a reference assembly, and Mangot et al.^24^ recovered about 20% of the genome when assembling cells of the protist group MAST-4. However, both studies highlighted the importance of amplifying the DNA from several cells, as this greatly increased recovery^23,24^.

A promising method to reduce bias associated with MDA, and thereby increase genomic coverage, is to divide the amplification reaction into nano-sized droplets, a method referred to as droplet MDA (dMDA)^25–27^. The idea behind dMDA is to isolate the target DNA fragments into tiny droplets and thereby reducing the competition of encountering a polymerase, leading to a more uniform amplification and overall improved genome coverage. Marcy et al.^25^ tested the effect of droplet MDA by detection of 10 gene loci from 14 dMDA and 12 standard MDA reactions of *E. coli* cells and found that all 10 loci were found in all 14 dMDA samples, but that several loci were missing from multiple standard MDA samples. In addition, samples generated with dMDA displayed a much more uniform amplification (measured by copies of loci/ul) than the samples generated by standard MDA. Likewise, the genome recovery from sequencing *E. coli* cells was increased from 59% with standard MDA to 89% using dMDA^28^.

The goal of the present study was to genome sequence and *de novo* assemble the genome of *A. acetabulum*. To obtain sufficient genomic DNA for sequencing we have amplified DNA from single embryonic cells using dMDA. We present an assessment of the sequencing data produced by single-cell dMDA, and its usefulness for assembly of large eukaryotic genomes. In addition, we compare three different assembly strategies; assembling each single-cell dMDA library separately, assembling these individual assemblies into a meta-assembly, and assembling all the sequencing libraries combined (co-assembly). This study is among the first to use single-cell dMDA for sequencing and *de novo* assembly of a eukaryote genome and should serve as a useful reference for future attempts to sequence species that are difficult to cultivate or collected from the environment.

## Methods

### Culturing *Acetabularia acetabulum* embryos

Embryonic cells of *A. acetabulum* were kept at 20 °C in culture flasks (T-25 filter cap tissue culture flasks from Sarstedt, Germany) with ca. 60 ml Dasycladales Seawater Medium prepared after the recipe of UTEX Culture Collection of Algae at The University of Texas at Austin (https://utex.org/products/dasycladales-seawater-medium?variant=30991770976346). Embryos were prevented from growing by storing the cells in the dark where they enter dormancy^29^. The medium was changed every three months, and at this time point the embryos were exposed to light (intensity of 45 μmol m^−2^ s^−1^) for 30 min.

### Harvesting *A. acetabulum* embryos and sonication of DNA

**Figure 1:**
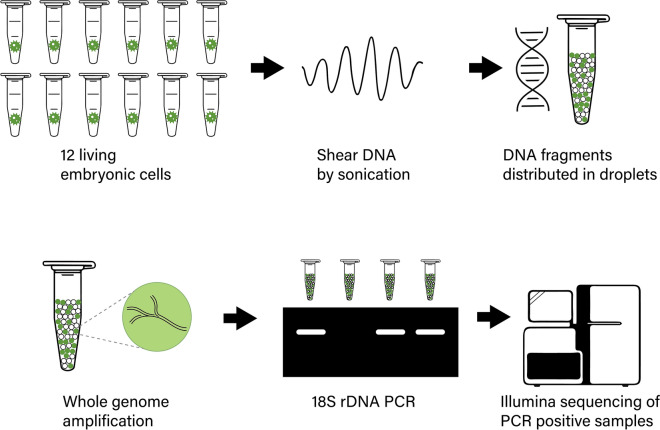
Schematic overview of the wet-lab procedure. 12 individual embryonic cells of *A. acetabulum* were isolated individually. The genomic DNA of the cells was shared using sonication before the DNA and multiple displacement amplification reagents were dispersed into thousands of micro-droplets. The amplification of genomic DNA was then performed inside these droplets before the droplets were merged and the DNA was prepared for sequencing and sequenced on the Illumina platform. See text for more details. Image credits: DNA Helix icon by Frey Wazza and Eppendorf tube icons by Julie Ko from the Noun Project.

Twelve embryos were washed three times in 10X PBS by manual suction pipetting under a Nikon Eclipse TS100 inverted light microscope and distributed into separate microTUBE-15 tubes (Covaris, MA, USA). Each microtube contained about 15 μl 10X PBS. The cells were lysed and DNA was fragmented by ultrasonication with an E200 Focused-ultrasonicator (Covaris) with a peak intensity power of 18, duty factor of 20%, cycles per burst of 50, and temperature of 20 °C (following the standard protocol). Half of the samples were sonicated for one minute and the remaining half were sonicated for three minutes. See Fig. 1 for a schematic overview of the lab workflow.

### Droplet MDA

A QX200 Droplet Generator (BIO-RAD, CA, USA) in combination with Pico-Surf 1 (2% in Novec 7500) oil (Dolomite Bio, UK) was used for droplet generation. MDA was performed according to protocol 1: *Amplification of genomic DNA from single cells* from the REPLI-g Single Cell Kit (Qiagen, MD, USA), with minor modifications to be compatible with the droplet generator; Since the standard sample input to the Droplet Generator is 20 μl, each sonicated cell/DNA sample (which contained 15 μl) was separated into two equal samples and each mixed with components from the REPLI-g Single Cell Kit to a total of 21 μl. 20 μl of this reaction was loaded on to the Droplet Generator and mixed with 40 μl Picro-Surf 1 (2% in Novec 7500) oil. After droplet generation, the amplification was run at 30 °C for eight hours. The emulsion was then broken and the single amplified genomes (SAGs) were recovered following the “Amplicon Recovery from Droplets” protocol (BIO-RAD).

### Confirmation of *A. acetabulum* in the dMDA products

PCR amplification of the ribosomal 18S rDNA gene by the use of the universal eukaryotic primers 1F (5′-CTGGTTGATCCTGCCAG-3′)^30^ and EUK1134R (5′-TTTAAGTTTCAGCCTTGCG-3′)^31^ was performed on all twelve samples in order to verify the presence of genomic DNA from *A. acetabulum*. The amplification was performed with the Green Taq Master Mix (Qiagen) and using an amplification protocol of an initial denaturation at 94 °C for 2 min, then 35 cycles of 94 °C for 30 s, 52 °C for 30 s, and 72 °C for 1 min, with a final extension of 72 °C for 10 min. The PCR products were verified on a 1% agarose gel. Samples with a single band of the expected size (ca. 1.7–1.8 kb) were cut from the gel and purified using Wizard® SV Gel and PCR Clean-Up System Protocol (Promega Corp., WI, USA). Purified PCR products were Sanger sequenced by GATC BIOTECH (Ebersberg, Germany). *A. acetabulum* 18S rDNA sequences were identified by BLASTn^32^ searches against the nr/nt Nucleotide Collection using the NCBI web server.

### Illumina sequencing of dMDA products and sequence processing

Five SAGs containing the *A. acetabulum* 18S rDNA gene were prepared into sequencing libraries suitable for the Illumina HiSeq4000. One full lane on the flow cell was used and 150 bp paired-end sequences were generated. The library preparation and sequencing were done at the Norwegian Sequencing Centre, Oslo, Norway (www.sequencing.uio.no).

Remnants of the 3′-adapter sequences were removed using Trim Galore v/0.3.3 (https://github.com/FelixKrueger/TrimGalore), followed by an additional trimming with Trimmomatic v/0.35^33^, which removed remaining adapter sequences, low-quality bases (Phred score cut-off of 20), and sequences shorter than 20 bp after trimming.

### Removing non-*A. acetabulum* sequence reads

Kraken2 v/2.0.8^34^ was used to assign a taxonomic classification to the Illumina reads. The trimmed reads were compared to a database consisting of all bacterial, archaeal, and eukaryote genomes and sequences in the NCBI non-redundant nucleotide collection (downloaded using Kraken2) as well as chloroplast genomes of 12 Ulvophycean species from Turmel et al.^35^.

### Genome assembly

#### Assembly of individual SAGs

Reads classified by Kraken2 as “root” or “Eukaryota” (meaning that they contain sequences conserved across a very broad taxonomic range), “Viridiplantae” (including all reads classified within Viridiplantae) as well as “unclassified” reads were considered to be potential *A. acetabulum* reads and were used in the assemblies. Individual SAG assemblies were constructed from the trimmed reads using SPAdes v/3.13.1^36^ with the –sc option for single-cell (MDA) assembly and *k*-mer sizes of 33, 55, 77, 99, and 121.

#### Meta-assembly of SAG assemblies

The contigs of the resulting SAG assemblies were assembled into a meta-assembly (an assembly of the assemblies) with the hybrid long-read assembler in Geneious (Geneious Prime 2019.2) (www.geneious.com). The greedy assembly-algorithm first starts with a blast-like algorithm to find the closest matching sequences for each contig among all the other contigs (using an initial word length of 24 to initiate the match, a minimum overlap of 125 bases between contigs, a maximum mismatch of 5%, and a maximum of 5% gaps). The highest scoring contig pairs are merged into a new contig. The process is repeated iteratively, either joining contigs or appending additional contigs where necessary, creating successively longer scaffolds. The resulting meta-assembly is composed of both consensus sequences constructed by contigs from two or more SAG assemblies (hereafter referred to as scaffolds), and contigs from the individual SAG assemblies that did not have any overlap with contigs from another SAG assembly.

#### Co-assembly of all sequence libraries

A co-assembly of the trimmed sequences from all five SAG libraries together was constructed using SPAdes v/3.10.1^36^ with the –sc option and k-mer sizes of 33, 55, 77, 99, and 121.

Scaffolds or contigs shorter than 500 bp were removed from all assemblies before further analysis. Simple repeats were identified using RepeatMasker v/4.0.9^37^ with the -species option set to Viridiplantae. Summary statistics of the assemblies were produced by Assemblathon^38^. BUSCO v/3.0.2^39^ run against the Chlorophyta database was used to estimate genome completeness. In addition, 246,083 *A. acetabulum* transcriptome sequences (unpublished data from Andresen et al.) were mapped against all assemblies separately using Minimap2 v/2.17^40^ with the –splice option, to evaluate genome completeness based on the number of mapped transcripts. Only transcripts with a mapping quality ≥ 20 were considered successfully mapped. Venn diagrams of *A. acetabulum* transcripts mapping to the SAG assemblies, meta-assembly, and co-assembly were constructed using the systemPipeR package^41^ in R.

### Evaluation of amplification bias

#### Normalization based on sequencing depth

To estimate the proportion of duplicated reads in the sequencing libraries, the Illumina reads from each SAG library were normalized using BBNorm (included in the BBMap/v38.50b suite, https://sourceforge.net/projects/bbmap/). The script was set to output read files with an average read depth of 100x, and discard reads with reads depths below 5x.

#### Downsampling analysis

Subsampling and re-assembly of reads were performed to investigate heterogeneity and to estimate the need for deeper sequencing of the individual SAG libraries. SPAdes was used to generate sub-assemblies per SAG library based on 10–90% of randomly sampled reads (in 10% increments). As previously, the –sc option and k-mer sizes of 33, 55, 77, 99, and 121 were used as parameters during SPAdes assembly. The assembly sizes were plotted using R and ggplot2^42^.

#### Genomic coverage

Trimmed reads from each SAG library were mapped to the meta-assembly with BWA v/0.7.17^43^ allowing each read to map only to a single location. Coverage files were constructed using samtools depth^44,45^ printing all positions (option -aa) and maximum coverage depth of 1,000,000 (option -d 1,000,000). The coverage files were plotted using R and ggplot.

### Annotation with mapped transcriptome data

The transcripts that mapped against the meta-assembly were annotated by first merging all overlapping and adjacent (with no space in between) transcripts into a “pseudo-loci” using BEDTools v/2.30.0^46^. This was done to reduce redundancy in form of very similar transcripts and to have transcribed regions of the genome represented as single units (i.e. pseudo-loci). Each pseudo-locus was searched against the NCBI Reference RNA sequences database (refseq_rna) with a minimum e-value threshold of 0.001.

## Results

### Amplification and sequencing of genomic DNA from *A. acetabulum* using droplet MDA

All twelve embryo samples were successfully amplified, and ten of these single amplified genomes (SAGs) had PCR products of the expected size for the 18S rDNA gene (~ 1700 bp). Five SAGs (i.e. SAG10, SAG14-16, and SAG20) had an unambiguous amplification of the *A. acetabulum* 18S rDNA gene and were therefore selected for Illumina sequencing (Table 1).

**Table 1:** Amplification of genomic DNA from *A. acetabulum* embryonic cells using dMDA. Successful dMDA product was determined if high-molecular weight DNA was clearly visible on an agarose gel. **“**fwd” and “rev” sequence refers to PCR products sequenced with the 1F and EUK1134R primers respectively (see "Methods"). These sequences were considered to be of “good” quality if the sequencing chromatogram showed a clear and for the most part unambiguous sequence, and “poor” if otherwise. “Poor” sequences were not Blasted as no reliable sequence could be generated.

Sample	Successful dMDA product	PCR product of expected size	Quality of fwd sequence	Quality of rev sequence	Blast hit fwd sequence (Genbank accession nr)	Blast hit rev sequence (Genbank accession nr)
SAG9	Yes	Yes	Good	Good	Acetabularia 18S (Z33461)	No hit
SAG10	Yes	Yes	Good	Good	Acetabularia 18S (Z33461)	Acetabularia 18S (Z33461)
SAG11	Yes	Yes	Poor	Good	–	No hit
SAG12	Yes	Yes	Poor	Good	–	No hit
SAG13	Yes	No	–	–	–	–
SAG14	Yes	Yes	Good	Good	Acetabularia 18S (Z33461)	Acetabularia 18S (Z33461)
SAG15	Yes	Yes	Good	Good	Acetabularia 18S (Z33461)	Acetabularia 18S (Z33461)
SAG16	Yes	Yes	Good	Good	Acetabularia 18S (Z33461)	Acetabularia 18S (Z33461)
SAG17	Yes	Yes	Poor	Good	–	No hit
SAG18	Yes	Yes	Poor	Good	–	No hit
SAG19	Yes	No	–	–	–	–
SAG20	Yes	Yes	Good	Good	Acetabularia 18S (Z33461)	Acetabularia 18S (Z33461)

In total, approximately 364 million read pairs were generated from the five sequenced SAGs, with ~ 56–92 million pairs from each (Table 2). All reads were of high quality and more than 95% of the reads from each SAG were retained after quality filtering. More than 70% of the trimmed read pairs were classified as potential *A. acetabulum* reads based on Kraken2 and were used as input to the assemblies. The remaining ~ 30% of the reads were classified mainly as bacterial (15%), archaeal (4%), and metazoan (9%) and were discarded.

**Table 2:** Illumina sequencing of genomic DNA from *A. acetabulum* amplified using droplet MDA. Quality trimming reduced the number of paired reads by 3–5% per SAG. Between 67–76% of the read pairs per sample were classified by Kraken2 as “unclassified”, root (excluding subclasses), Eukaryota (excluding subclasses), or Viridiplantae (including subclasses) and were considered as potential *A. acetabulum* sequences (numbers above the dashed line). These reads were used as input to the genome assemblies. Below the dashed line are shown the most abundant groups of non-*Acetabularia* reads classified by Kraken2.

	SAG10	SAG14	SAG15	SAG16	SAG20
Raw sequence pairs	88,859,965	91,981,739	62,789,260	56,617,044	63,119,731
Sequence pairs after quality filtering (% reduction)	85,954,036 (3%)	88,571,138 (4%)	60,239,555 (4%)	53,917,700 (5%)	60,557,218 (4%)
**Kraken 2**					
Unclassified	67%	60%	56%	60%	66%
Root (excluding subclasses)	0.13%	0.12%	0.11%	0.13%	0.14%
Eukaryota (excluding subclasses)	0.15%	0.12%	0.11%	0.14%	0.16%
Viridiplantae	2.40%	2.10%	1.90%	2.30%	2.70%
Bacteria	15%	21%	32%	24%	18%
Archaea	4.10%	6.90%	0.82%	3.10%	0.58%
Metazoa	9.30%	7.80%	7.70%	8.30%	9.90%
Read pairs after Kraken (input to assemblies) (% reduction)	62,089,629 (28%)	67,677,814 (24%)	42,628,780 (29%)	36,106,491 (33%)	45,903,295 (24%)

### Genome assembly

Independent assembly of the five different SAG libraries resulted in assemblies of total sizes from ~ 42 to ~ 59 Mb, with an N50 ranging from ~ 2 to ~ 5 Kb (Table 3). The different assemblies varied in the number of detected Chlorophyta BUSCO genes from 152 (7%) to 198 (9%), and of mapped *A. acetabulum* transcripts from 1418 (1%) to 15,916 (6%).

**Table 3:** Comparison of the genome assemblies using three different assembly strategies. SAG10-20 refers to the separate assemblies of the different SAG read libraries. Meta-assembly is the assembly of the five SAG assemblies. Co-assembly is the assembly of the five SAG libraries pooled. Scaffolds or contigs shorter than 500 bp are removed from all assemblies and the calculation of the N50s. Contigs/scaffolds refers to either contigs in the and SAG- and Co-assemblies, or scaffolds in the meta-assembly.

	SAG10	SAG14	SAG15	SAG16	SAG20	Meta-assembly	Co-assembly
Input read pairs	62,089,629	67,677,814	42,628,780	36,106,491	45,903,295	–	254,406,009
Genome coverage*	10	11	6.7	5.5	7.3	–	40
Total size of contigs/scaffolds (bp)	58,977,243	52,998,455	53,810,482	59,345,876	42,658,079	147,591,821	118,824,433
N50 contig/scaffold length	2750	2566	5646	5102	3954	6209	4481
#Contigs/scaffolds	32,637	30,449	20,455	24,489	19,840	55,499	53,141
Longest contig/scaffold (bp)	445,368	343,663	294,237	320,419	343,769	739,579	187,706
GC-content (%)	47	50	52	51	50	50	50
Simple repeats (%)	0.26	0.22	0.22	0.29	0.20	0.27	0.29
Mapped transcripts	15,916 (6%)	5717 (2%)	1418 (1%)	10,368 (4%)	2914 (1%)	18,047 (7%)	18,219 (7%)
Contigs/scaffolds with mapped transcripts	2574 (8%)	935 (3%)	520 (3%)	848 (3%)	632 (3%)	2898 (5%)	3201 (6%)
Chlorophyta BUSCOs	152 (7.0%)	152 (7.0%)	198 (9.1%)	189 (8.7%)	161 (7.4%)	180 (8.3%)	241 (11%)
Complete and single-copy	107 (4.9%)	110 (5.1%)	142 (6.5%)	130 (6.0%)	124 (5.7%)	93 (4.3%)	168 (7.7%)
Complete and duplicated	34 (1.6%)	34 (1.6%)	38 (1.8%)	44 (2.0%)	20 (0.9%)	65 (3.0%)	53 (2.4%)
Fragmented	11 (0.5%)	8 (0.4%)	18 (0.8%)	15 (0.7%)	17 (0.8%)	22 (1.0%)	20 (0.9%)

*Genome coverage was calculated by the total nr of input bases to each assembly divided by the estimated size of the diploid *A. acetabulum* genome (1764Mb).

Assembling the individual SAG assemblies into a meta-assembly increased the assembly size to ~ 148 Mb (which is about 8% of the estimated diploid genome size of 1764 Mb^47^), and the N50 to 6209 bp. Despite these improvements, the number of detected Chlorophyta BUSCO genes and mapped transcripts did not increase as 180 (8%) Chlorophyta BUSCOs genes were found and 18,047 (7%) of *A. acetabulum* transcripts mapped to the meta-assembly.

Pooling the five SAG libraries and assembling them to a co-assembly gave a total assembly size of ~ 119 Mb (6.74% of the diploid genome) distributed on 53,141 scaffolds with an N50 of 4481 bp. 241 (11%) Chlorophyta BUSCO genes were detected, and 18,219 (7%) of the *A. acetabulum* transcripts mapped to the co-assembly.

Assuming that genes and expressed transcripts are evenly distributed along the *Acetabularia* genome, the BUSCO results and the mapping of *A. acetabulum* transcripts suggest that we have recovered approximately 7–11% of the *A. acetabulum* genome. This estimation also corresponds roughly with the total sizes of the meta- and co-assemblies.

**Figure 2:**
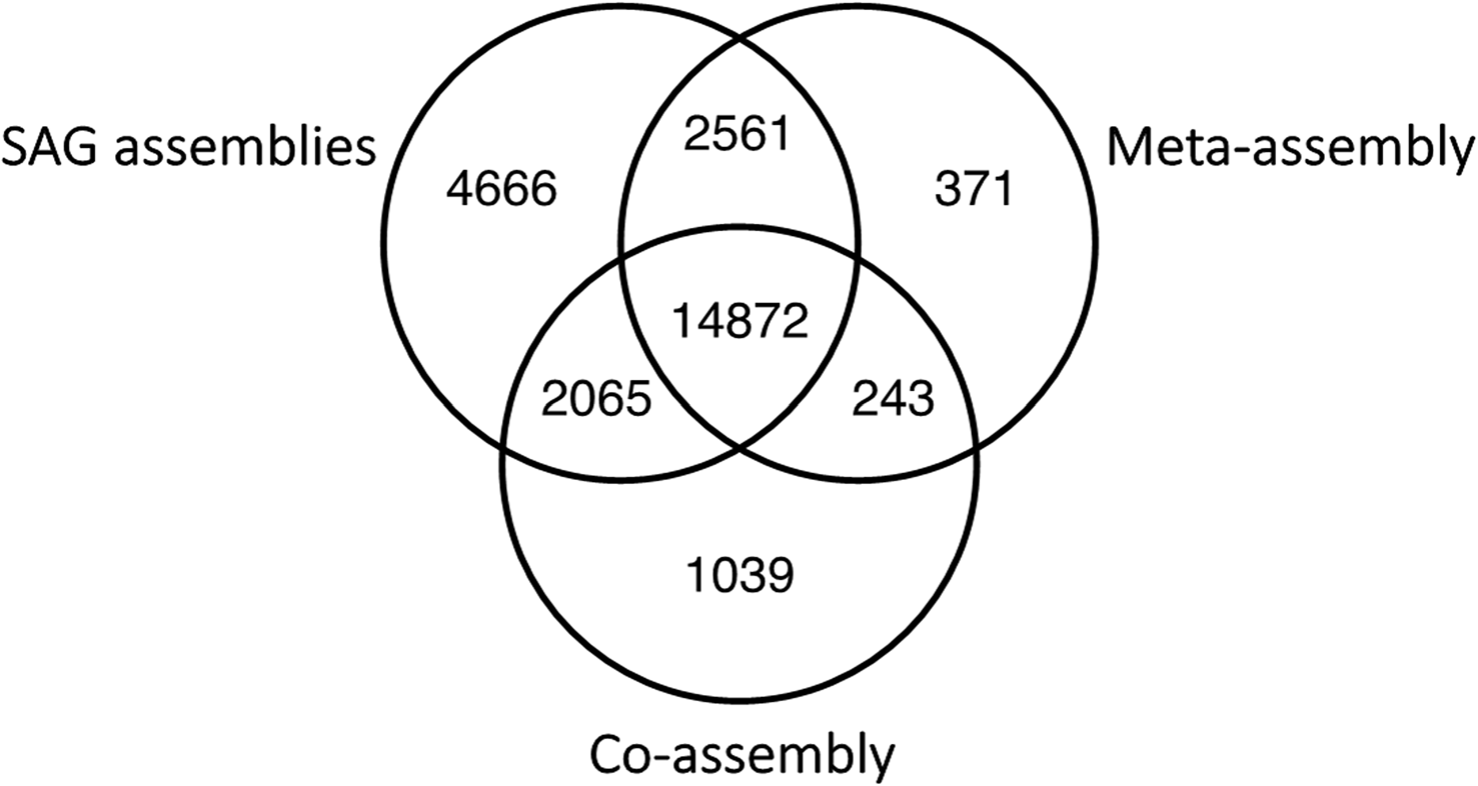
Comparison of the transcripts mapping to the assemblies. Venn diagram (drawn using the systemPipeR package^41^ in R) showing the similarities and differences of the *A. acetabulum* transcripts mapping to the individual SAG assemblies (transcripts mapping to the five SAG assemblies are combined), Meta-assembly, and Co-assembly.

Comparing the number of *A. acetabulum* transcripts mapping to the different assemblies (Fig. 2) shows that 14,872 transcripts mapped to at least one of the SAG assemblies (transcripts mapping to the five SAG assemblies are combined), as well as to both the meta- and co-assembly. 4666 transcripts mapped only to the SAG assemblies, 371 transcripts mapped only to the meta-assembly, while 1039 transcripts mapped only to the co-assembly.

### Investigating the effect of amplification bias

**Figure 3:**
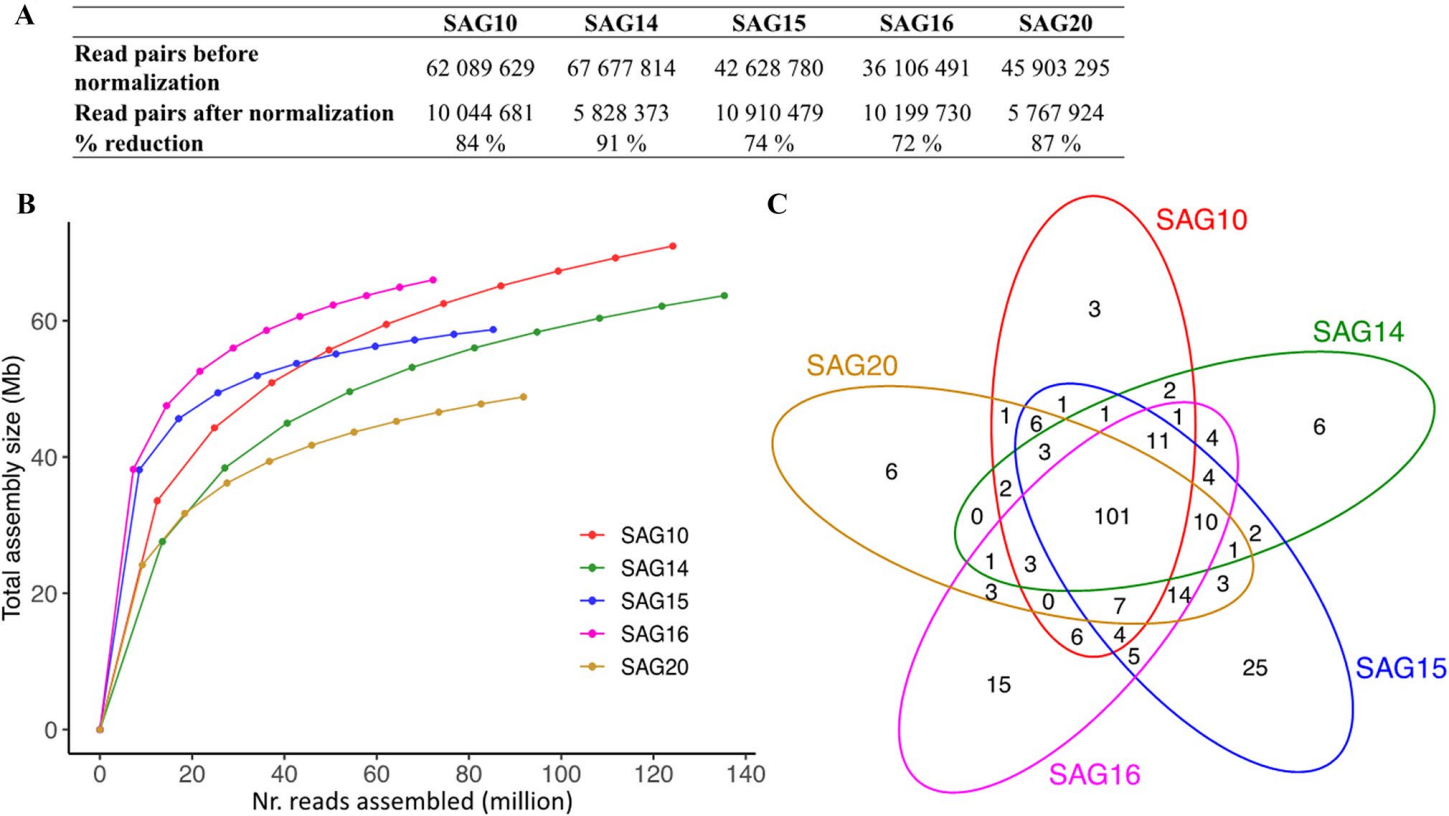
The effect of amplification bias on individual dMDA samples. (**A**) Number of paired Illumina reads before and after normalization with BBNorm. (**B**) Down-sampling analysis (of non-normalized reads) showing the assembly size (in megabases) on the y-axis and the number of paired reads used as input for the assembly on the x-axis. Plot created with the ggplot2^42^ R package. (**C**) Venn diagram (drawn using the systemPipeR package^41^ in R.) comparing the detected Chlorophyta BUSCO genes in each SAG assembly.

Normalizing reads to an even coverage of *k*-mers reduced the number of reads drastically and removed 72–91% of the reads per sample (Fig. 3A), indicating that a high level of duplicated reads was sequenced. Assemblies of subsampled reads (not normalized) from individual SAGs showed that the assembly sizes increased with an increasing number of input reads, but also showed signs of stabilizing when all the reads were used for the assembly (Fig. 3B). The majority of the detected Chlorophyta BUSCO genes (101 genes) were found in all five SAG assemblies, while between 3 and 25 BUSCO genes were uniquely found in each SAG assembly (Fig. 3C).

**Figure 4:**
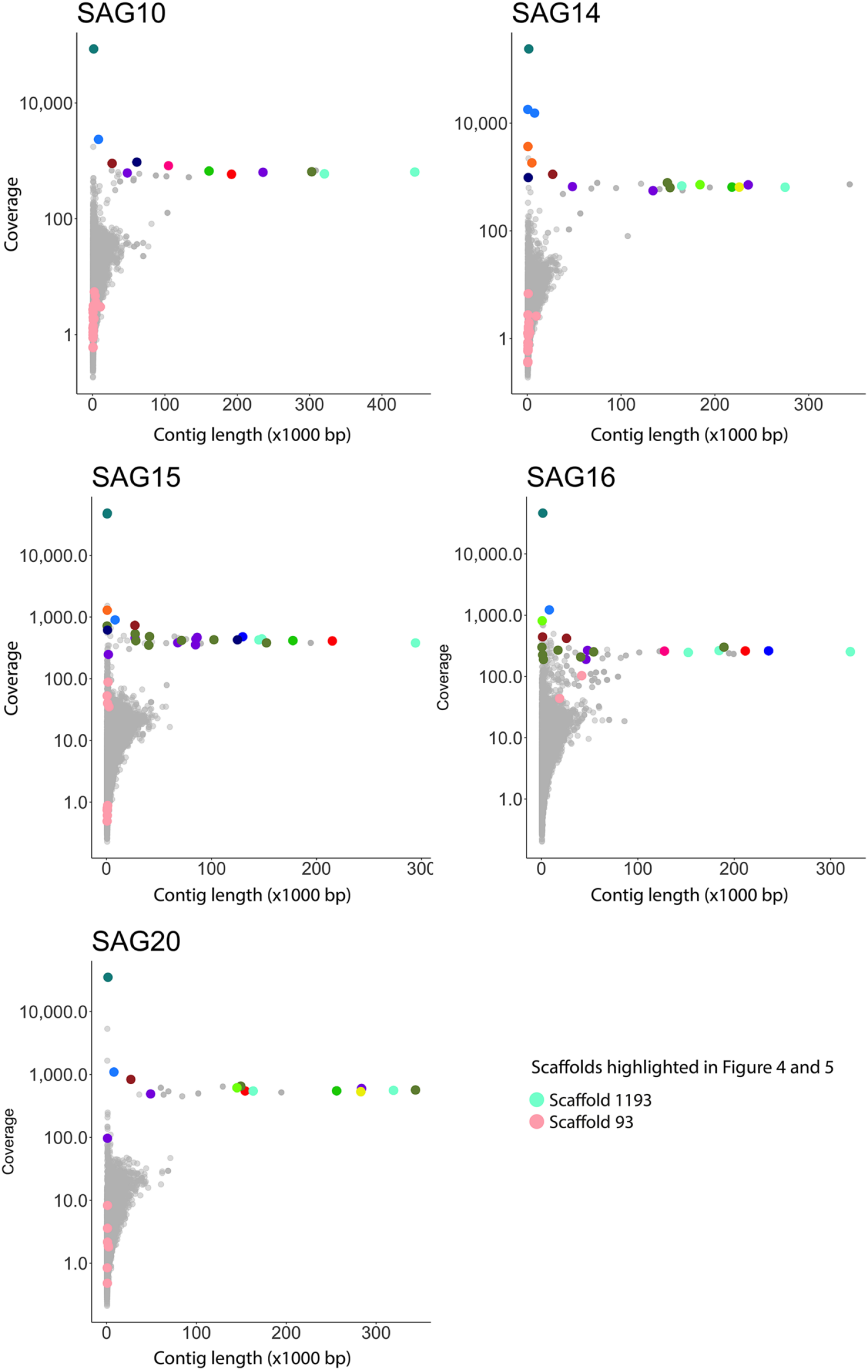
Coverage versus length of individual SAG assemblies. The *k*-mer coverage for each contig in the SAG assemblies as reported by the SPAdes assembler is shown on the y-axis and the length of the contig (in thousand bp) is shown on the x-axis. A selection of the longest contigs, and the contigs with the highest coverage (i.e. highly amplified genomic regions) are colored according to which genomic region they originate from (i.e. are assembled into the same scaffold in the meta-assembly). E.g. the contig with the highest coverage in all SAGs is from the same genomic region (dark blue color), and some of the longest contigs in almost all SAGs are from the same genomic region (aquamarine color; Scaffold 1193 in the figure legend). Scaffold 93 is also highlighted (pink color) as this portion of the genome is almost exclusively amplified in SAG16 and is in this sample assembled into two rather long contigs which make up scaffold 93 in the meta-assembly. This genomic region is therefore only represented by very small and low coverage contigs in the other SAGs. Plots created with the ggplot2^42^ R package.

Plotting the coverage of the contigs from the individual SAG assemblies versus their lengths (Fig. 4) showed that aside from a few short contigs, the longest contigs were also the ones with the highest coverage, indicating solid and high-quality sequencing and assembly of these contigs. Interestingly, most of these long contigs have been assembled into the same scaffolds in the meta-assembly (as indicated in Fig. 4). The contig with the highest coverage was the same sequence in all SAG assemblies and has been assembled into the same scaffold in the meta-assembly (i.e. scaffold 3799). In general, the shorter contigs have overall low coverage (Fig. 4).

**Figure 5:**
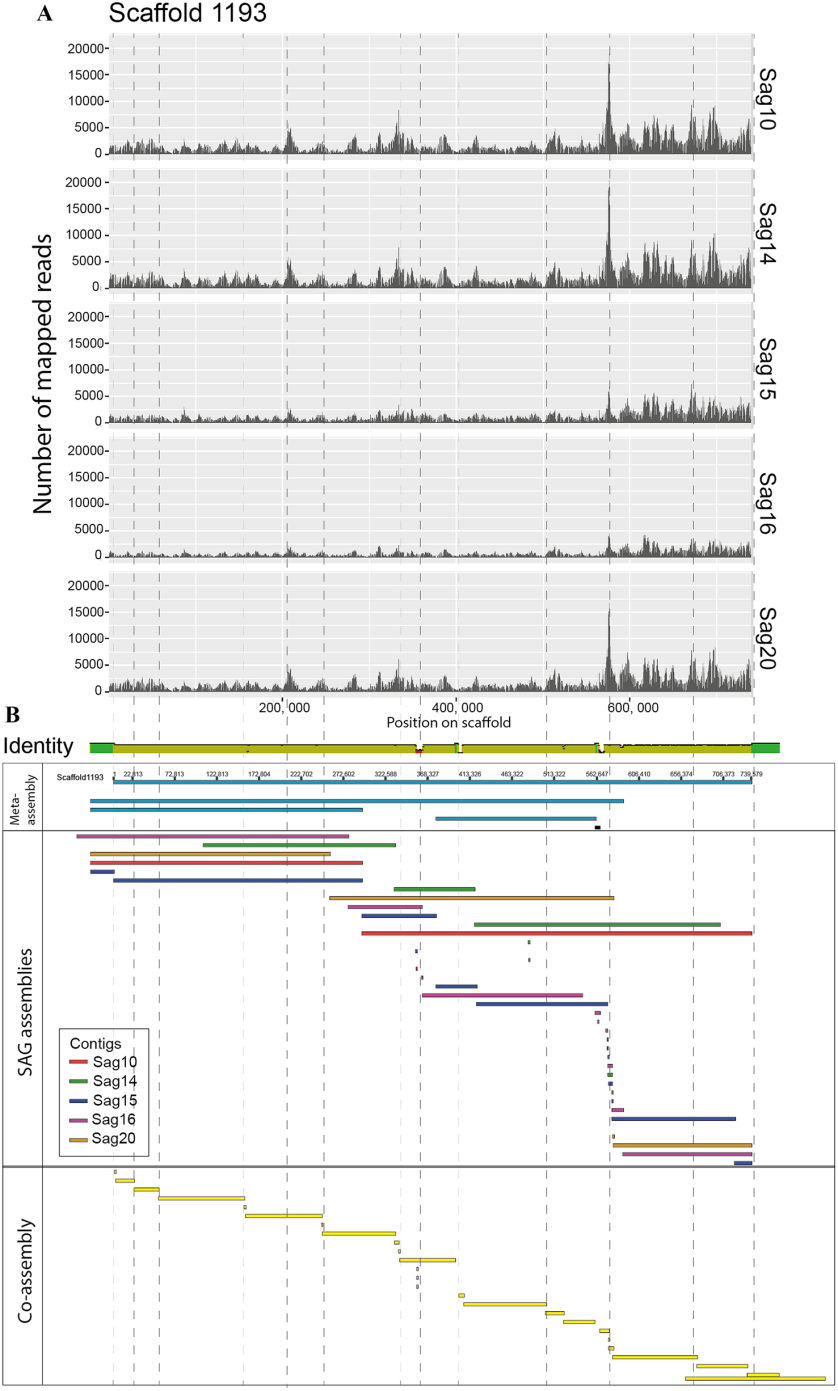
Genome coverage and alignment of contigs to scaffold 1193 in the meta-assembly. (**A**) Mapped Illumina reads from each SAG library to scaffold 1193 from the meta-assembly. (**B**) An alignment of the contigs from the individual SAG assemblies and the contigs from the co-assembly against scaffold 1193. Vertical lines align the peaks in coverage from panel (**A**) to breakpoints in contigs of the co-assembly in panel (**B**). Plots created with the ggplot2^42^ R package and Geneious Prime 2019.2 (www.geneious.com).

The longest scaffold in the meta-assembly (scaffold 1193, Fig. 5A) was also assembled into some of the longest contigs in each of the SAG assemblies (marked in aquamarine blue Fig. 4). Investigating the mapping of reads to this scaffold shows that this region of the genome has been highly and evenly amplified in all five samples. This is a good example of even amplification of the same genomic region in all five dMDA samples, but could also indicate systematic amplification bias between samples. It also illustrates how the meta-assembly strategy is able to connect individual contigs into longer scaffolds. There are other scaffolds with high similarity to scaffold 1193 in the meta-assembly, but with a few smaller regions of either mismatches or insertions and deletions that prevent them from being included in the longest scaffold. Whether these mismatches represent assembly artefacts, or allelic variations of the same genomic region, cannot be determined with the present data, but we see that in the co-assembly contigs, many shorter contigs align perfectly to meta-scaffold 1193. Interestingly, the breaks in the co-assembly correlate with sudden shifts in the coverage of highly amplified genomic regions (either a peak or a valley between two peaks, vertical lines in Fig. 5), which is probably the reason why the SPAdes assembler has not been able to connect these regions into a single contig. Most likely, the different SAG contigs depicted in Fig. 5B should have been merged into a single scaffold.

**Figure 6:**
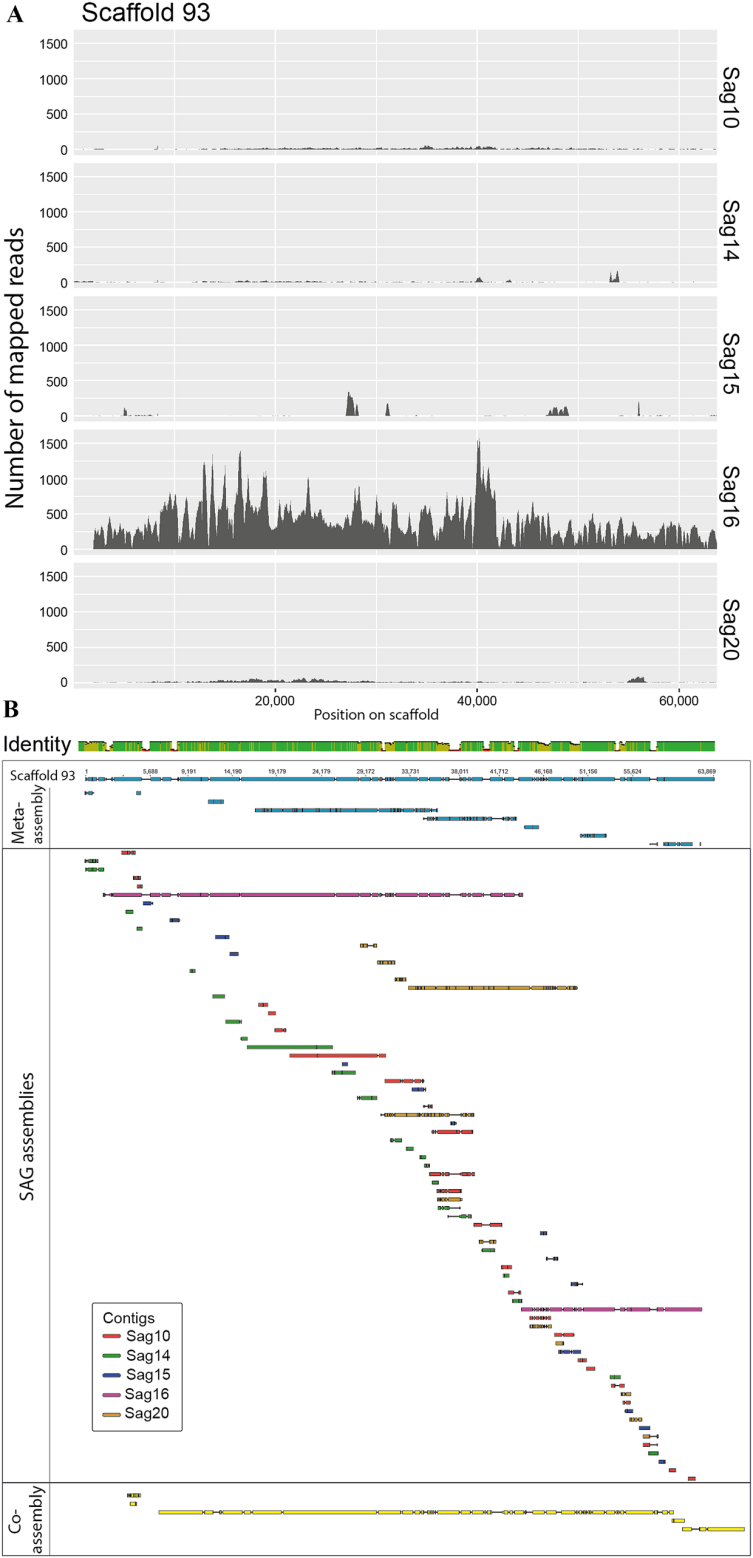
Genome coverage and alignment of contigs to scaffold 93 in the meta-assembly. (**A**) Mapped Illumina reads from each SAG library to scaffold 93 from the meta-assembly. (**B**) An alignment of the contigs from the SAG assemblies against scaffold 93. The alignment also contains contigs from the co-assembly aligned to the same scaffold. This scaffold represents a portion of the genome almost exclusively amplified in SAG16. See also the description in the text. Plots created with the ggplot2^42^ R package and Geneious Prime 2019.2 (www.geneious.com).

Scaffold 93 represents a region of the genome almost exclusively amplified in SAG16 (Fig. 6A). The genomic region has only to a small extent been amplified in the other SAGs, and the alignment of contigs to the scaffold (Fig. 6B) shows that this region has been assembled into many short and low coverage contigs (also indicated in pink color in Fig. 4). This genomic region was recovered in the co-assembly as five different fragments (Fig. 6B).

### Annotation of transcribed regions

The 18,047 transcripts that mapped to the meta-assembly (Table 3) were merged into 6286 different “pseudo-loci” (i.e. overlapping and adjacent transcripts) distributed across 2898 scaffolds. The pseudo-loci covered in total 2,627,134 bp (ca. 1.8% of the meta-assembly), had a mean and median length of 419 and 260 bp. The highest number of pseudo-loci located on a single scaffold was 49 (Contig_2) with 42 (Contig_8) and 40 (Contig_41) as the second and third highest (the mean and median number was 2.2 and 1). Only 313 pseudo-loci had a significant Blast hit. Among these, chloroplast, mitochondria, and ribosomal genes were among the most abundant. In addition to several bacterial and eukaryote unannotated genomic hits (Supplementary Table 1).

## Discussion

### Using dMDA to amplify and assemble the genome of *A. acetabulum*

This study provides the first report of sequencing and assembling the nuclear genome of *Acetabularia acetabulum*. We have done this through whole genome amplification using multiple displacement amplification in microfluidic droplets (dMDA) of five embryos. Even though MDA has previously been used to amplify eukaryote genomes^5,23,24,48–53^, it has not yet been used to amplify eukaryote genomes as large as the *A. acetabulum* genome. In addition, MDA combined with microfluidic droplets has until now only been used to amplify bacterial genomes^25–27^.

### The dMDA assembly is highly fragmented and consists of large gene deserts

Not surprisingly, the most genetic information was found in the assemblies based on all the different samples. These varied in size from 119–148 Mb and we were able to identify around 7% of the *A. acetabulum* expressed loci and identify around 10% of the genes included in the Chlorophyta BUSCO database. Altogether, these results support that we have been able to recover approximately 10% of the *A. acetabulum* genome.

Because of the low genome recovery and the highly fragmented assemblies we were not able to perform any robust annotation of the genome. Mapping of expressed transcripts suggests that less than 2% of the genome is expressed, which is not unexpected for a large eukaryote genome. However, we were surprised that we could identify homologs of only a tiny fraction of the expressed loci. And quite remarkably, none of the longest scaffolds had any mapped transcripts even though several are close to 1 Mb long. This could indicate that the vast majority of the *A. acetabulum* genome is gene deserts of non-coding and non-transcribed DNA. The genome of *Caulerpa lentillifera*, another macroscopic single-celled Ulvophyte^54^ is in comparison only 26 Mb^55^ and much more gene dense. The extremely large genome size of *A. acetabulum* could therefore be a result of expansions of non-coding and intergenic regions rather than gene duplications and innovations. Although both *C. lentillifera* and *A. acetabulum* are macroscopic single-celled algae, they differ in cell physiology in that *C. lentillifera* is a syncytium with potentially thousands of nuclei scattered along the cell^55^, while *A. acetabulum* only has a single nucleus. It is therefore tempting to speculate that the differences in genome size between the two species are related to their differences in cell biology and demand for production and transport of nuclear transcripts.

### More samples are needed to recover the full *A. acetabulum* genome

There are in general two strategies to increase the amount of genomic information recovered from single-cell MDA: increasing the sequencing depth and increasing the number of amplified genomes. Even though all SAG assemblies contained some unique portions of the genome, the downsampling analysis shows that a deeper sequencing of the samples is not likely to recover more of the genome. In addition, deeper sequencing will lead to much more sequences to handle computationally and only increase the uneven coverage along the genome. It is therefore probably more fruitful to increase the number of samples, or amplified cells. As exemplified by Scaffold 93, which represented a large portion of the genome only amplified in a single sample, the genome amplification between samples was still highly uneven even though the reaction was distributed across thousands of droplets. Increasing the number of samples is therefore more likely to increase the genome recovery.

### Differences in sensitivity and specificity between the meta-assembly and the co-assembly

López-Escardó et al.^23^ and Mangot et al.^24^ showed that pooling sequences from independent SAGs resulted in higher genome recovery. However, in a recent study using MDA on single fungal cells, Montoliu-Nerin et al.^53^ showed that they in fact got a larger genome size and higher number of scaffolds by performing a meta-assembly of individual assemblies. Still, these studies were performed on very different protist species, with different genome characteristics. We tested and evaluated the resulting assemblies from three assembly approaches; assembling high-quality reads into five separate SAG assemblies, scaffolding the assembled contigs from the five SAG assemblies to a meta-assembly, and assembling reads from all the five SAGs together into a co-assembly. Assembling each SAG separately resulted in highly fragmented and very small assemblies, most likely due to the very low genomic coverage of each individual sample. Even though nearly the same number of transcripts mapped to each SAG assembly as to the meta- and co-assembly, most of these transcripts mapped either at the ends of short contigs (with a large portion of the transcripts extending outside the contig), or mapped with only parts of the transcript matching the contig (possible chimeric contigs or assembly artefacts), which highlights the problems of assembling each amplified sample separately.

Combining and assembling all the reads into a co-assembly nearly doubled the size compared to each SAG assembly (in total base pairs) and gave a higher number of mapped transcripts and detected BUSCOs. However, the assembly was still highly fragmented. The longest contig was shorter than for any of the SAG assemblies, and the N50 was shorter than for two of the SAG assemblies. Alignments between contigs from the individual SAG assemblies and the co-assembly also clearly demonstrated that several of the long contigs in the individual SAG assemblies were not reproduced in the co-assembly, indicating that the assembler has not managed to construct contigs from such uneven coverage.

On the other hand, the meta-assembly had the largest total size and longest N50, statistics often associated with a higher assembly quality. Many of the shorter contigs in the co-assembly had been assembled into longer scaffolds in the meta-assembly, probably because the meta-assembler does not rely on read coverage but the overlap and identity of contigs, and hence is not affected by the highly uneven coverage introduced by the MDA. Nevertheless, the meta-assembly had fewer mapped transcripts and detected BUSCOs than the co-assembly. In addition, many scaffolds should probably have been merged into a single scaffold, hence the total size and number of scaffolds is probably inflated in the meta-assembly.

The choice of assembly approach depends on the downstream use of the assembly. The meta-assembly resulted in the longest scaffolds and the largest total assembly size and could be useful in for instance large-scale comparisons such as studies of synteny and genome evolution. However, if accuracy of the individual sequences is more important, then the co-assembly strategy is more suitable.

### MDA bias is a major challenge, even with droplet amplification

To reduce the amplification bias and increase the overall genome recovery, we distributed the MDA reaction across thousands of microfluidic droplets. Still, there was a significant amount of amplification bias in our datasets. Normalizing the sequence reads based on the depth of coverage (i.e. removing identical or very similar reads) showed that the vast majority were duplicated, meaning that they originate from a few highly amplified regions of the genome. The high number of duplicated reads also means that we in practice had a much lower genome coverage than the theoretical estimates, which to a large extent explains the poor assembly and genome recovery. The MDA was performed on the genomic DNA directly after cell lysis and fragmentation, without any purification in between. This was done to minimize loss of DNA, but it also means that the DNA could still have been tightly packed into chromatin at certain places, which probably has increased the amplification bias. Reducing the amplification time of the MDA could be one strategy to avoid high numbers of duplicated sequences, as the most amplified genomic regions will become preferentially amplified over time. Removing organellar DNA is probably also important as circular DNA can be expected to be preferentially amplified.

In addition to amplification bias within each sample, the different SAGs were also quite similar in sequence content in that almost the same BUSCOs were detected in each sample and many scaffolds had reads mapping from each sample. This indicates that there was also a non-random amplification of certain genomic areas in each SAG. This bias between samples could have been introduced by our strategy of only sequencing MDA products where we could amplify the *A. acetabulum* 18S rDNA gene. This is a common strategy when doing MDA on cells collected from the environment^24^. However, this might have favored samples with similar amplification patterns and DNA content. Unsuccessful PCR amplification of the 18S rDNA gene does not necessarily mean that *A. acetabulum* DNA was not present in that sample but could also be a result of the amplification bias. Therefore, using more genetic markers to verify the presence of the target species in the MDA reaction should be used in future experiments.

## Supplementary Information

Supplementary Information.

## Data Availability

All sequence data and genome assemblies generated in this study have been deposited to the European Nucleotide Archive under the study accession PRJEB40379.
